# Mitochondria in neurodegenerative diseases

**DOI:** 10.1111/cns.13183

**Published:** 2019-06-14

**Authors:** Fang Lin, Shou‐Qing Luo

**Affiliations:** ^1^ Department of Pharmacology, Laboratory of Aging and Nervous Diseases (SZS0703) Soochow University School of Pharmaceutical Science Su Zhou China; ^2^ Peninsula Schools of Medicine and Dentistry, Institute of Translational and Stratified Medicine University of Plymouth Plymouth UK

The mitochondrion consists of 3000 proteins. 3% (about 100 proteins) among them are responsible for creating 95% ATP needed for cells. The remaining 97% of proteins take part in non‐ATP‐related functions. Mitochondria have long been known as “energy factories,” and now, we are expanding the knowledge from the biology and structure of mitochondrion to diseases. In the central nervous system (CNS), in addition to ATP production, mitochondrial proteins are also important for the metabolism of neurotransmitters, reactive oxygen species (ROS) production, calcium homeostasis, and cell death/survival regulation. The dysfunctional mitochondrion has been involved with aging, pain, and neurodegenerative diseases in CNS.

Enhancing the biogenesis of functional mitochondria or removing dysfunctional mitochondria may be a potential approach to retard the aging process and treat the related diseases. Yan Wang and Erin Xu at University of North Carolina at Chapel Hill, Phillip R. Musich at East Tennessee State University, and Fang Lin at Soochow University reviewed mitochondrial dysfunction in aging, neurodegenerative diseases, and the potential countermeasure.^1^ Compounds, which enhance mitochondrial biogenesis, reduce ROS levels, and induce mitophagy, would restore mitochondrial homeostasis in aging and neurodegenerative diseases. Caloric restrict and exercise modulate mitochondrial biogenesis, activating mitophagy for removal of damaged mitochondria. Exercise could enhance autophagy‐lysosomal pathway and decrease mitochondrial vacuolization caused by chloroquine through restoring autophagic flux.^2^, ^3^

Mitochondrial fission and fusion impact numerous cellular functions, and neurons are particularly sensitive to perturbations in mitochondrial dynamics. Katrina Cowan, Oleg Anichtchik, and Shouqing Luo at Plymouth University highlighted the current knowledge in the fundamentals of mitochondrial biology and discussed the mitochondrion‐related cytotoxicity, mitochondrial fission and fusion dynamics, and mitophagy‐mediated mitochondrial quality control.^4^ The authors also summarized distinct mitochondrial dysfunctions in neurodegenerative diseases, focusing on Parkinson's disease, Huntington's disease, and Alzheimer's disease.

Proteins posttranslational modifications including nitrosylation, SUMOylation, and phosphorylation regulate the activity and function of the protein. Protein kinases and phosphatases can migrate to the outer mitochondrial membrane and mediate the signals between the mitochondrion and the cell body, and therefore control the structure and function of mitochondria. Drp1 translocates from the cytosol to the outer mitochondrial membrane to initiate its fission. PKA‐mediated phosphorylation of Drp1 inhibits its fission activity, whereas dephosphorylation by the Ca2+‐dependent phosphatase calcineurin (PP2B) or PP2A activates the enzyme. AKAP1/PKA protects against cerebral ischemic stroke by inhibiting Drp1‐dependent mitochondrial fission.^5^ Maribel Lucero, Ana E. Suarez, and Jeremy W. Chambers at Florida International University summarized the effects of kinases, phosphatases, and the associated adaptor proteins and scaffold proteins on the OMM in neurons.^6^ The authors reviewed the roles of specific substrate phosphorylation or dephosphorylation events including the impacts on mitochondrial dynamics, cell death, fission/fusion, and metabolism. The deficiency in the kinase or phosphatase activities associated with the pathophysiology of neurological diseases was also discussed.

Dysfunctional mitochondria can be degraded by mitophagy, which is crucial to maintain mitochondrial healthy. PINK1 and Parkin have been identified as central players in mitophagy. Bingwei Lu's laboratory at Stanford University recently found that OXPHOS mRNAs stalled on the damaged mitochondrial surface to form the mRNA‐ribonucleoprotein complex. The cotranslational quality control factors Pelo, ABCE1, and NOT4 are recruited to the mRNA‐ribonucleoprotein complex, and ABCE1 is ubiquitinated by NOT4. As a result, the poly‐Ub‐ABCE1 recruits the autophagy receptors such as p62, PTEN, and NDP52 to initiate mitophagy.^7^ In this issue, Yan Wang and Na Liu at Soochow University and Bingwei Lu reviewed the mechanisms of mitophagy in higher organisms and the roles of mitophagy in the pathogenesis of neurodegenerative diseases.^8^ Although mitophagy is critical to safeguard cell homeostasis, excessive or reduced mitophagy may be harmful to cells.

Mitochondria can be transported along and anchored in axons and synapses with the change of axonal and synaptic physiology,^9^ but the lysosomes mainly locate at the proximity of the nucleus. It was puzzling how mitochondria in distal axons are cleared through mitophagy. Zhong Chen's laboratory at Zhejiang University observed that axon‐derived mitochondria appeared in neuronal soma and underwent autophagic clearance. Anchoring of axonal mitochondria by syntaphilin blocked neuronal mitophagy and aggravated injury after OGD and reperfusion. Conversely, induced binding of mitochondria to dynein reinforced retrograde transport and enhanced mitophagy to prevent mitochondrial dysfunction and attenuate neuronal injury. Their findings provide a new concept to alleviate ischemic neuronal injury by correcting mitochondrial motility.^10^ In this issue, the authors, Yanrong Zheng, Xiangnan Zhang, and Zhong Chen, reviewed the process of mitochondrial transportation.^11^ The newly generated mitochondria are anterogradely transported from soma to the axon, and the damaged mitochondria undergo retrograde transport for repair or autophagic clearance. The abnormal transport of mitochondria leads to the accumulation of dysfunctional mitochondria and the degeneration of neurons in neurological and psychiatric diseases. Notably, N‐terminal mutant huntingtin impairs mitochondrial trafficking.^12^ In the meanwhile, endolysosomal deficits result in the mitochondrial pathology. Expressing dynein‐adaptor snapin, which reverses transport defects and rescues autophagy‐lysosomal deficits, enhanced mitochondrial turnover and improved the survival of motor neurons.^13^

Recently, J. Marie Hardwick's laboratory at Johns Hopkins University has discovered that KCTD7 mutations lead to the defect in autophagy‐lysosomal pathway. Patients with KCTD7 mutations exhibit movement disorders or developmental regression before seizure onset. The electron microscopy showed abnormal mitochondrial cristae morphologies, lipid droplet accumulation around mitochondria, and phagolysosomes containing partially degraded material in KCTD7‐mutant patient fibroblasts.^14^ In this special issue, Xinchen Teng at Soochow University, Abdel Aouacheria and Loïc Lionnard at Université de Montpellier, and Kyle A. Metz, Lucian Soane, Atsushi Kamiya, and J. Marie Hardwick at Johns Hopkins University reviewed the biological functions of KCTD family proteins (such as potential roles in potassium channels and protein quality control) and their involvement in neurodevelopmental and neuropsychiatric disorders.^15^ Undoubtedly, the molecular insights will facilitate our understanding in the complexity of neurodevelopment and neurology diseases.

Mitochondrial biology plays an increasingly important role in neuroscience research. Over recent years, significant advancement has been made in the areas including mitochondrial dynamics, bioenergetics, fission/fusion, metabolism, and mitophagy. However, more biochemical mechanisms, for example in mitochondrial homeostasis and mitophagy, yet await elucidation, particularly those relevant to mitochondrial diseases and neurodegenerative conditions. The current special issue highlights a few critical advances in the biological processes of mitochondria and neurological diseases. Dissemination of the knowledge is a necessary step toward the path to uncovering the enigmas underlying mitochondrial homeostasis and related diseases.

Lastly, we would like to thank all the authors, reviewers, and the editorial office for their great contributions to this special issue.
